# Tunable Thermo-Responsive Properties of Hydroxybutyl Chitosan Oligosaccharide

**DOI:** 10.3389/fchem.2022.830516

**Published:** 2022-03-10

**Authors:** Chong Chen, Weibo Zhang, Yan Zhang, Pengjie Wang, Fazheng Ren

**Affiliations:** ^1^ Key Laboratory of Functional Dairy, Co-constructed By Ministry of Education and Beijing Government, College of Food Science and Nutritional Engineering, China Agricultural University, Beijing, China; ^2^ Department of Nutrition and Health, China Agricultural University, Beijing, China; ^3^ College of Food Science and Engineering, Gansu Agricultural University, Lanzhou, China

**Keywords:** hydroxybutyl chitosan oligosaccharide, thermoresponsive, lower critical solution temperature, hydrophobic interaction, hydrogen bond

## Abstract

In this study, a simple method was used to synthesize novel thermosensitive hydroxybutyl chitosan oligosaccharide (HBCOS) by introducing hydroxybutyl groups to C_6_–OH of chitosan oligosaccharide (COS) chain. The variation in light scattering demonstrated that HBCOS had good thermosensitive properties and the particle size of HBCOS changed from 2.21–3.58 to 281.23–4,162.40 nm as the temperature increased to a critical temperature (LCST). The LCST of HBCOS (10 mg/ml) decreased from 56.25°C to 40.2°C as the degrees of substitution (DSs) increased from 2.96 to 4.66. The LCST of HBCOS with a DS of 4.66 decreased to 33.5°C and 30°C as the HBCOS and NaCl concentrations increased to 50 mg/ml and 4% (w/v), respectively. Variable-temperature FTIR spectroscopy confirmed that dehydration of hydrophobic chains and the transition of hydrogen bonds were the driving forces for the phase transition of HBCOS. Moreover, HBCOS was not cytotoxic at different concentrations. This work generated a novel thermosensitive HBCOS with tunable thermoresponsive properties and excellent biocompatibility, which may be a potential nanocarrier for the biomedical application.

## Introduction

Over the past decades, environment-sensitive materials have gained extensive attention because of their controllable shrinkage or swelling behaviors in response to specific physicochemical stimuli including temperature, pH, light, and ionic strength (Chen et al., 2017; Ji et al., 2017; Yu et al., 2018; Theune et al., 2019). The stimuli-responsive properties of these materials make them ideal platforms for drug delivery as they can release the entrapped drug at the appropriate time and location. Among various stimuli, temperature is one of the most widely investigated for smart drug delivery as the stimuli can be induced by intracellular or remote-controlled thermal changes (Simpson et al., 2018).

Numerous synthetic thermo-responsive materials including poly (N-isopropylacrylamide) (PNIPAAm), poly (N,N-diethylacrylamide) (PDEAAm), poly (ethylene oxide), and poly (N-vinlycaprolactam) (PVCL) have been developed over the years for biomedical applications (Ward and Georgiou, 2011; Zhang et al., 2016). The phase transition behavior of such synthetic polymers is controlled by the proportion, chain length, molecular weight, and architecture of copolymerizable hydrophilic or hydrophobic monomers (Ward and Georgiou, 2011). However, the application of these synthetic thermosensitive polymers are limited due to their nonbiocompatibility or nonbiodegradability (Qiu and Park, 2001; Qureshi et al., 2019). Thus, developing biocompatible and biodegradable thermoresponsive materials is necessary for the application of thermo-sensitive polymers in the biomedical field.

Recently, modification of natural macromolecules to prepare biocompatible and biodegradable thermoresponsive biopolymers has gained much more attention (Bi et al., 2020; Sun et al., 2020). Hydroxybutyl derivatives of chitosan and hydroxypropyl derivatives of cellulose have been synthesized by introducing hydrophobic groups to polysaccharide chains, which exhibits excellent temperature-responsive properties, biocompatibility, and biodegradability (Dang et al., 2006; Qi et al., 2012; Hu et al., 2018; Li et al., 2020). However, a complicated preparation, including purification with acid and freeze (below −10°C)–thawing process (under high alkaline/urea conditions), is required to dissolve these polysaccharides in NaOH prior to carrying out further alkylation modifications (Qi et al., 2012; Bi et al., 2017). Furthermore, the high alkalinity of the reaction system results in the degradation of the polysaccharide chains (Sun et al., 2017; Cai et al., 2019). Thus, the poor solubility of natural macromolecules in alkaline condition limits their alkylation modification.

Chitosan oligosaccharides (COS, degree of polymerization <20) are derived from chitosan via chemical and enzymatic hydrolysis processes (Naveed et al., 2019). COS can be dissolved directly under neutral or alkaline conditions without additional preprocessing steps due to its shorter chain length and low level of intermolecular interactions (Muanprasat and Chatsudthipong, 2017). More importantly, COS has several reactive functional sites for chemical modification, including secondary hydroxyl groups at C6 and an amine/acetamide group at C2 (Naveed et al., 2019). Therefore, COS is more appropriate for modification as an optional biopolymer to generate an original thermoresponsive biomaterial than chitosan and cellulose. In this situation, introducing hydrophobic groups (hydroxybutyl groups) into the COS chain may generate a novel thermosensitive material, of which the process of alkylation could avoid complicated preparation steps and high alkalinity that is needed for chitosan and cellulose. Surprisingly, no studies have focused on the alkylation modification of COS to synthesize COS-based thermosensitive materials.

Thus, the aim of this study was to synthesize thermoresponsive hydroxybutyl chitosan oligosaccharide (HBCOS) by conjugating hydroxybutyl groups of 1,2-butylene oxide to COS chain and investigate the thermoresponsive behaviors and mechanism of HBCOS. Here, the successful synthesis of HBCOS was proven by Fourier transform infrared spectroscopy (FTIR) and hydrogen nuclear magnetic resonance (^1^H NMR). The thermoresponsive behaviors of HBCOS were investigated by dynamic light scattering (DLS), TEM, and static multiple light scattering. The changes in the chemical groups in interaction and conformation during the phase transition were determined by *in situ* FTIR to explore thermoresponsive mechanisms of HBCOS. This work provides a simple and stable method to synthesize thermosensitive HBCOS, thus, developing a potential nanocarrier for drug delivery.

## Materials and Methods

### Materials

Chitosan oligosaccharide (Mw = 1,811 Da, DD = 91.5%) was bought from Weikang Biomedical Technology Co., Ltd. (Shandong, China). 1,2-Butylene oxide was purchased from TCI Shanghai Development Co., Ltd. (Shanghai, China). Deuterium oxide solution was purchased from Sigma Aldrich (St. Louis, MO, USA). All other chemical reagents used in the study were of analytical grade.

### Preparation of Hydroxybutyl Chitosan Oligosaccharide

A 1% (w/w) COS solution was prepared by dissolving 1 g of COS in 99 g of ultrapure water under continuous stirring. NaOH was added to a final concentration of 0.2 mol/L. Then different volumes (12.5, 15, 17.5, 20, and 22.5 ml) of 1,2-epoxy butane were added to the above COS solution and stirred constantly at room temperature for 48 h. The mixture was neutralized with 4 mol/L of HCl to stop the reaction and dialyzed (regenerated cellulose dialysis bag, MWCO 500 Da) with ultrapure water for 2 days. The mixture was then lyophilized to obtain HBCOS.

For convenience, HBCOS samples with different degrees of substitution (DSs) obtained from the reaction between 1 g of COS and 12.5, 15, 17.5, 20 and 22.5 ml of 1,2-epoxy butane were labeled as HBCOS-12.5, HBCOS-15, HBCOS-17.5, HBCOS-20, and HBCOS-22.5, respectively.

### Characterization of Hydroxybutyl Chitosan Oligosaccharide

The FTIR spectra of HBCOSs and COS were recorded ranging from 4,000 to 400 cm^−1^ using a Bruker INVENIO S FTIR spectrometer (Bruker, Germany) equipped with a Gladi-ATR accessory (Pike Technologies, Madison, WI, USA). ^1^H NMR spectra of HBCOSs and COS were recorded on a Bruker AVANCE III 600 MHz (Bruker, Germany) at 25°C to measure the DS. The DS of HBCOSs was calculated using the following equation (Cai et al., 2019):

$$\text{DS}=\frac{{\text{S}}_{{\text{CH}}_{3}}/3}{{\text{S}}_{{\text{H}}_{1}}}$$

Herein, ${\text{S}}_{{\text{H}}_{1}}$ is the integral area of the peaks attributed to the H_1_ protons, and ${\text{S}}_{{\text{CH}}_{3}}$ is the integral area of the peaks attributed to the hydroxybutyl methyl protons.

### Thermoresponsive Behaviors of Hydroxybutyl Chitosan Oligosaccharide Solution

Static multiple light scattering and DLS were used to investigate the thermoresponsive behaviors of HBCOS. Static multiple light scattering was performed at different temperatures using Turbiscan LAB (Formulation, Toulouse, France). HBCOS aqueous solutions (10 mg/ml) with different DSs, and different concentrations of HBCOS-20 (1–50 mg/ml) were poured into cylindrical glass tubes to be scanned every 12 min. Before being scanned, the temperature was raised and maintained to equilibrate the samples at a corresponding temperature. The LCST of HBCOS, which was defined as the temperature at an abrupt change in diameter, was measured by evaluating the size changes as a function of temperature (Yu et al., 2009). The particle size of HBCOS based on number mean was measured by DLS using a Zetasizer (Model Nano-ZS3600, Malvern, UK). Before the measurement, all samples were stabilized at the appropriate temperature for 600 s. The thermoresponsive behaviors of HBCOS-20 solutions (10 mg/ml) were observed using a TEM (JEM-1200EX, JEOL Ltd., Japan). The HBCOS-20 solutions were casted onto a holey copper grid-supported carbon film and dried at 35°C, 40°C, and 42°C, respectively, for the TEM observation.

### Thermoresponsive Mechanism of Hydroxybutyl Chitosan Oligosaccharide Solution

Variable-temperature FTIR spectroscopy was performed using a Bruker tensor 27 spectrometer (Bruker, Germany) to investigate the thermoresponsive mechanism of HBCOS solution. HBCOS-20 was dissolved in D_2_O (10 mg/ml) and pipetted into the middle of two CaF_2_ windows to collect spectrum information. The temperature of the IR cell was increased from 35°C to 41°C at a rate of 0.3°C/min. The analyses of IR spectra were carried out using the OMNIC 8.0 software.

### *In Vitro* Cytotoxicity of Hydroxybutyl Chitosan Oligosaccharide

The CCK-8 assay was performed to evaluate the *in vitro* cytotoxicity of HBCOS in Caco-2 cell lines (Wang et al., 2019). Briefly, Caco-2 cells were seeded in 96-well culture plates at a density of 2 × 10^4^ cells per well and cultured for 12 h. Then cells were treated with different concentrations of HBCOS-20 (5–0.1563 mg/ml). After incubation for 24 h, CCK-8 (10 μl) was added to each well and incubated for 1 h. The absorbance of HBCOS-treated and -untreated (control) samples was determined using a microplate reader (Infinite 200 Pro, Tecan, Austria) at 450 nm. Cytotoxicity was evaluated based on the relative cell viability with the control group set at 100%.

### Statistical Analysis

All experiments were carried out independently at least twice. The data were expressed as means ± standard deviations, which were calculated from these independent measurements. SPSS 20 was used for analysis of variance (ANOVA) by Tukey test, and *p* < 0.05 indicated significance.

## Results and Discussion

### Characterization of Hydroxybutyl Chitosan Oligosaccharide

**FIGURE 1:**
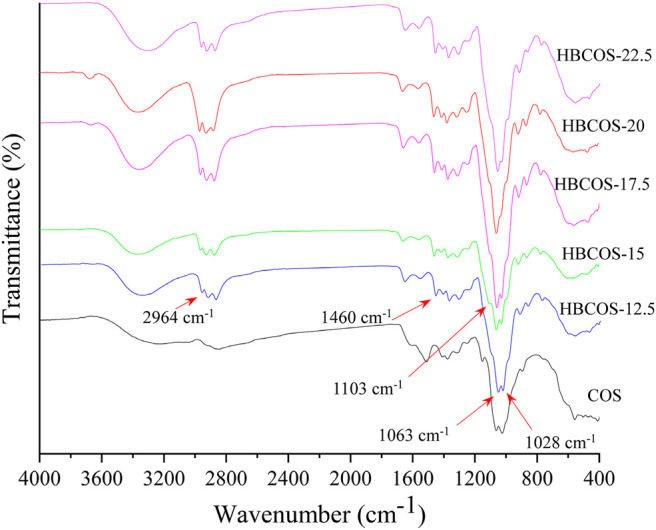
Fourier transform infrared spectroscopy (FTIR) spectra of chitosan oligosaccharide (COS) and hydroxybutyl chitosan oligosaccharides (HBCOSs).

The FTIR spectra of HBCOSs and COS are shown in Figure 1. Compared with the spectra of COS, additional peaks were observed in all HBCOSs at 2,964–2,878 and 1,460 cm^−1^, which were attributed to the stretching of C–H and bending of –CH_3_ groups in the hydroxybutyl groups (Jiang et al., 2019). These results indicated that the hydroxybutyl groups of 1,2-butylene oxide were successfully introduced to the COS chains. The absorption peak attributed to C–O stretching vibration of C_6_–OH in COS at 1,028 cm^−1^ was shifted to 1,063 cm^−1^ in HBCOSs after introducing the hydroxybutyl groups, indicating that hydroxybutyl substitution occurred at C_6_–OH (Jiang et al., 2019). Moreover, a new peak that could be attributed to ether bond at 1,103 cm^−1^ in HBCOS also appeared, which further proved that the substitution reaction of the hydroxybutyl group occurred at the C_6_–OH position (Li et al., 2020).

**FIGURE 2:**
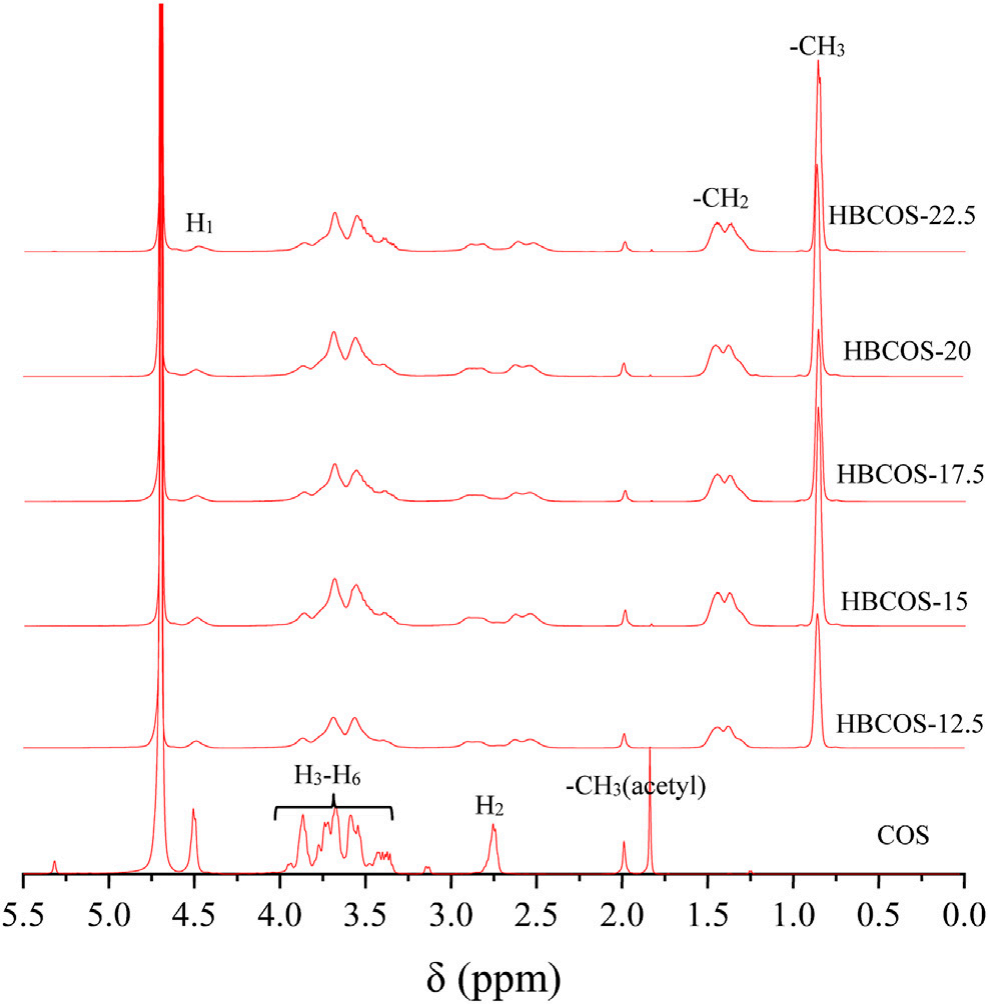
Hydrogen nuclear magnetic resonance (^1^H NMR) spectra of COS and HBCOSs.

The substitution reaction was also confirmed by ^1^H NMR. As shown in Figure 2, new peaks were observed in HBCOS at 0.8–1.5 ppm with the ratio of the integrated area being 3:2, which were not present in COS. Those peaks were attributed to the methyl and methylene of the hydroxybutyl groups (Cai et al., 2019). These results indicated that hydroxybutyl groups was introduced to the COS chains. The DS of HBCOS obtained from COS reacting with different volumes of 1,2-epoxy butane was calculated by the ratio of the integral area of hydroxybutyl methyl proton peaks to the H_1_ proton peaks (Table 1). The DS of HBCOS increased from 2.96 to 4.66 as the volume of 1,2-epoxy butane increased from 12.5 to 20 ml/g COS, showing a linear correlation between the DS and the volume of 1,2-epoxy butane. However, the DS decreased when the volume of 1,2-epoxy butane was higher than 20 ml/g COS. This may be attributed to the side reaction (self-polymerization of 1,2-epoxy butane in the presence of NaOH) and the change in solution polarity, which were induced by the excess 1,2-epoxy butane (Herzberger et al., 2016; Liu et al., 2021).

**TABLE 1:** Physicochemical properties of hydroxybutyl chitosan oligosaccharides (HBCOSs) with different degrees of substitution (DSs).^†^

	DS	LCST (°C)	Diameter (nm)	
			Before phase transition	After phase transition
HBCOS-12.5	2.96 ± 0.04	56.25 ± 0.96^d^	3.39 ± 0.21	586.67 ± 35.25
HBCOS-15	3.42 ± 0.20	46.50 ± 1.00^c^	2.76 ± 0.28	575.99 ± 38.78
HBCOS-17.5	4.22 ± 0.19	43.50 ± 0.58^b^	2.63 ± 0.59	587.77 ± 36.88
HBCOS-20	4.66 ± 0.10	40.20 ± 0.42^a^	3.46 ± 0.78	663.31 ± 33.51
HBCOS-22.5	4.21 ± 0.28	43.67 ± 0.52^b^	2.76 ± 0.74	624.76 ± 41.98

† The different superscript letters within the same column indicate significant difference (*p* < 0.05).

### Thermoresponsive Behaviors of Hydroxybutyl Chitosan Oligosaccharide

**FIGURE 3:**
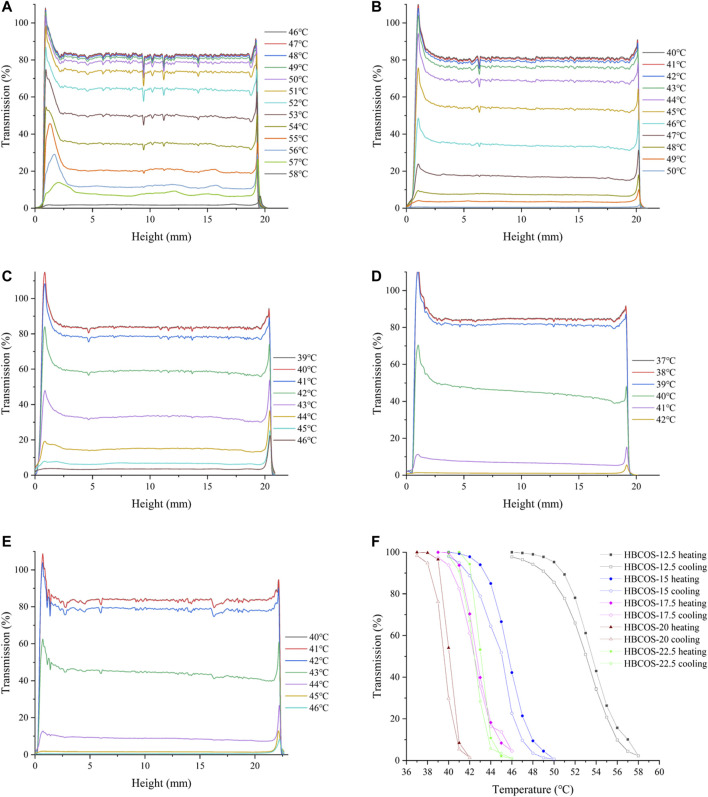
Changes in the transmission intensity profiles of HBCOSs as a function of temperature. **(A)** HBCOS-12.5, **(B)** HBCOS-15, **(C)** HBCOS-17.5, **(D)** HBCOS-20, **(E)** HBCOS-22.5. **(F)** Normalized transmission intensity of HBCOSs during heating–cooling process.

The optical properties of HBCOS solution were monitored as a function of temperature using Turbiscan Lab to obtain real-time information on the phase transformation process. The transmission intensity (%) of all HBCOS solutions decreased as the temperature increased (Figures 3A–E). In fact, the transmission intensity decreased slowly initially and then decreased sharply when the temperature exceeded specific critical temperatures. For comparison, the mean values of transmission intensity of HBCOSs with different DSs were normalized and are shown in Figure 3F. The cloud point was defined as the temperature at a 50% HBCOS transmission intensity (Kempe et al., 2011; Hiruta et al., 2015). The cloud points of HBCOS-12.5, HBCOS-15, HBCOS-17.5, HBCOS-20, and HBCOS-22.5 were 54°C, 46°C, 42.5°C, 40°C, and 43°C, respectively. Interestingly, the DS of HBCOSs showed a negative correlation with the cloud point. This can be explained by the enhancement of the hydrophobic interactions among the hydroxybutyl groups with increases in DS (Cai et al., 2019). The increase in the transmission intensity upon cooling indicated that the thermoresponsive behaviors of all HBCOS samples were reversible, which were not affected by the DS. However, a hysteresis loop appeared during the heating–cooling process, which was attributed to the imprecise revivification of hydrogen bonds and the slower rate of hydrophobic dissociation compared with hydrophobic association during the phase transition (Sun et al., 2008; Cai et al., 2019). These results demonstrated that introducing the hydroxybutyl groups to the COS chain made the HBCOS possess reversible thermoresponsive properties.

DLS was used to further confirm the thermoresponsive properties of HBCOSs. The particle sizes based on number mean and size distributions of HBCOSs varying with temperature are shown in Table 1 and Supplementary Figure S2. The particle size of all HBCOSs with different DSs changed from 2.63–3.46 to 575.99–663.31 nm as the temperature increased to a critical temperature (LCST). The aggregation of HBCOSs induced by temperature also indicated that HBCOSs were thermoresponsive. The small particle size of HBCOSs below the LCST was attributed to the amphiphilic structure (hydroxybutyl and acetyl, –NH_3_, and −OH) of the HBCOS, which resulted in the molecular chains forming 2.63–3.46 nm spherical coils. Similar results have been reported in hydroxybutyl chitosan and PVCL_63_-*b*-PDMAEMA_101_ (Karesoja et al., 2014; Bi et al., 2020); however, their diameters were larger than those of the HBCOSs. When the temperature increased to the LCST, the spherical coils aggregated and formed nanohydrogels with larger particle sizes. The phase transition process of HBCOSs may be caused by the break of hydrogen bonds and the enhancement of hydrophobic interaction (Spěváček et al., 2012; Cai et al., 2019). Furthermore, HBCOS-20 with the highest DS showed the lowest LCST, indicating that the density of hydroxybutyl groups significantly influenced the thermoresponsive properties of HBCOSs.

**FIGURE 4:**
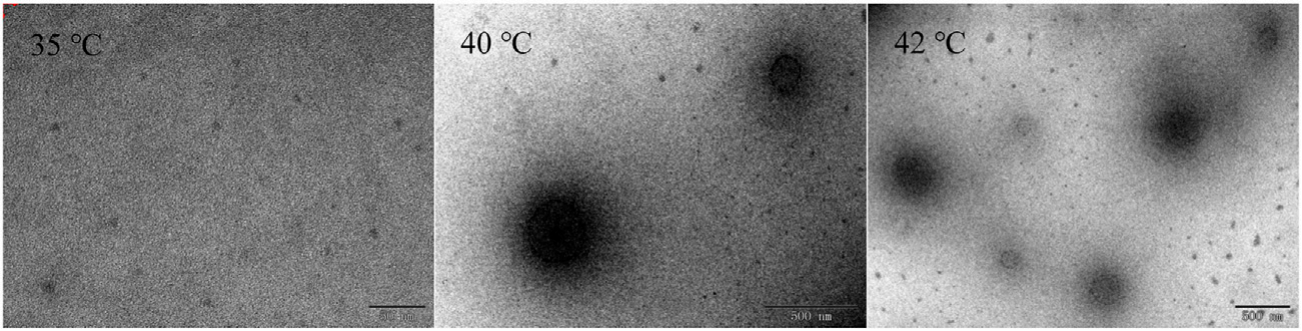
The TEM observation of HBCOS-20 solution (10 mg/ml) at different temperatures.

The phase transition process of HBCOS-20 was observed by TEM (Figure 4). The HBCOS-20 formed ∼5-nm spherical coils below LCST. The spherical coils aggregated and formed uniform and complete sphere as the temperature increased to LCST or above LCST. The particle size of HBCOS-20 from TEM after phase transition was smaller than the value from DLS due to the shrinkage of nanohydrogels during the sample preparation for TEM observation. These results confirmed the phase transition of HBCOS from spherical coils to nanohydrogels, which were consistent with the results in DLS.

**FIGURE 5:**
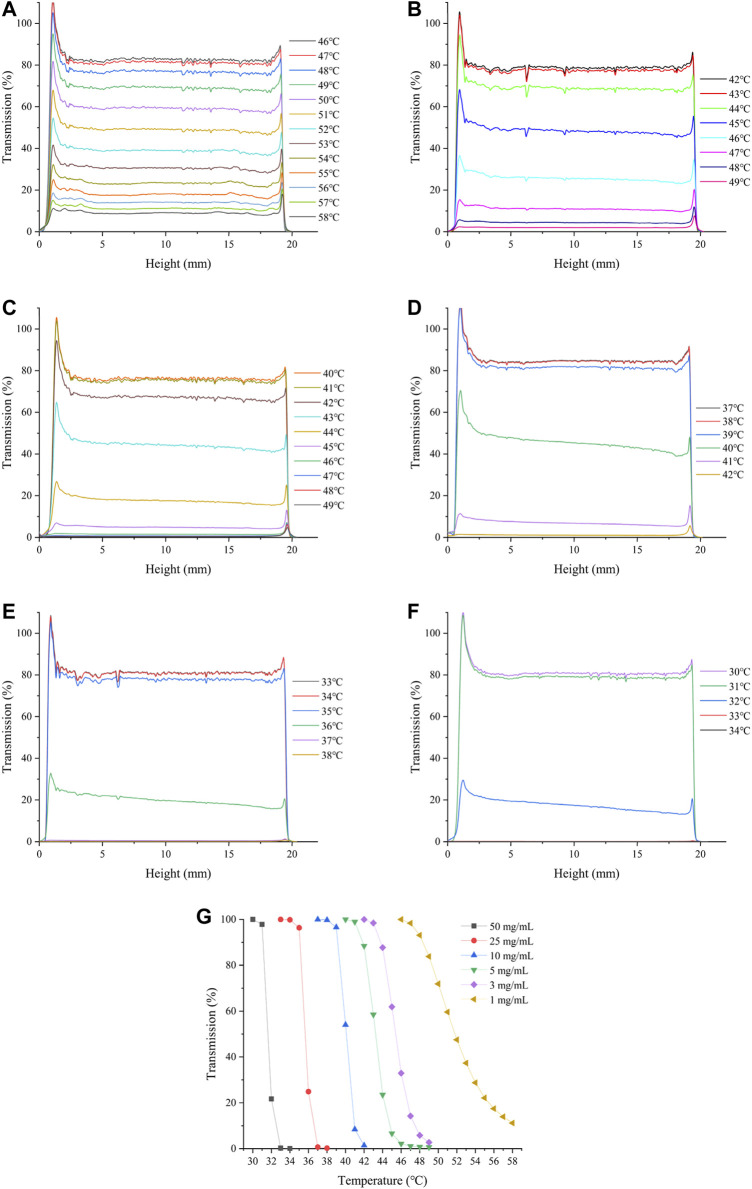
Changes in the transmission intensity profiles of HBCOS-20 with different concentrations as a function of temperature: **(A)** 1 mg/ml, **(B)** 3 mg/ml, **(C)** 5 mg/ml, **(D)** 10 mg/ml, **(E)** 25 mg/ml, **(F)** 50 mg/ml. **(G)** Normalized transmission intensity of HBCOS-20 with different concentrations.

The effect of HBCOS-20 solution concentration on its thermoresponsive behavior was investigated by static multiple light scattering and DLS. As shown in Figure 5 and Table 2, the LCST decreased from 47.67°C to 33.5°C as the concentration of HBCOS-20 increased from 1 to 50 mg/ml. This phenomenon indicated that the thermoresponsive properties of HBCOS could be adjusted by the solution concentration. Interestingly, HBCOS solution at higher concentrations exhibited more dramatic phase transition process and correspondingly larger particle sizes of the aggregated nanohydrogels. These results may be interpreted in terms of aggregation kinetics. Higher concentrations of HBCOSs with larger numbers of molecular chains per volume rendered it easier for collisions and aggregations to occur between the HBCOS chains (Ju et al., 2013; Tian et al., 2015).

**TABLE 2:** Influence of HBCOS-20 solution concentration on the LCST and particle size.^†^

Concentration (mg/ml)	LCST (°C)	Diameter (nm)	
		Before phase transition	After phase transition
1	47.67 ± 0.58^e^	3.29 ± 0.35	281.23 ± 28.95
3	44.50 ± 0.58^d^	2.88 ± 0.44	326.32 ± 40.66
5	43.25 ± 0.50^d^	3.05 ± 0.43	450.22 ± 22.28
10	40.20 ± 0.42^c^	3.46 ± 0.78	663.31 ± 33.51
25	37.25 ± 0.50^b^	3.00 ± 0.44	1,535.80 ± 76.08
50	33.50 ± 0.58^a^	2.95 ± 0.41	4,162.40 ± 215.1

† The different superscript letters within the same column indicate significant difference (*p* < 0.05).

**FIGURE 6:**
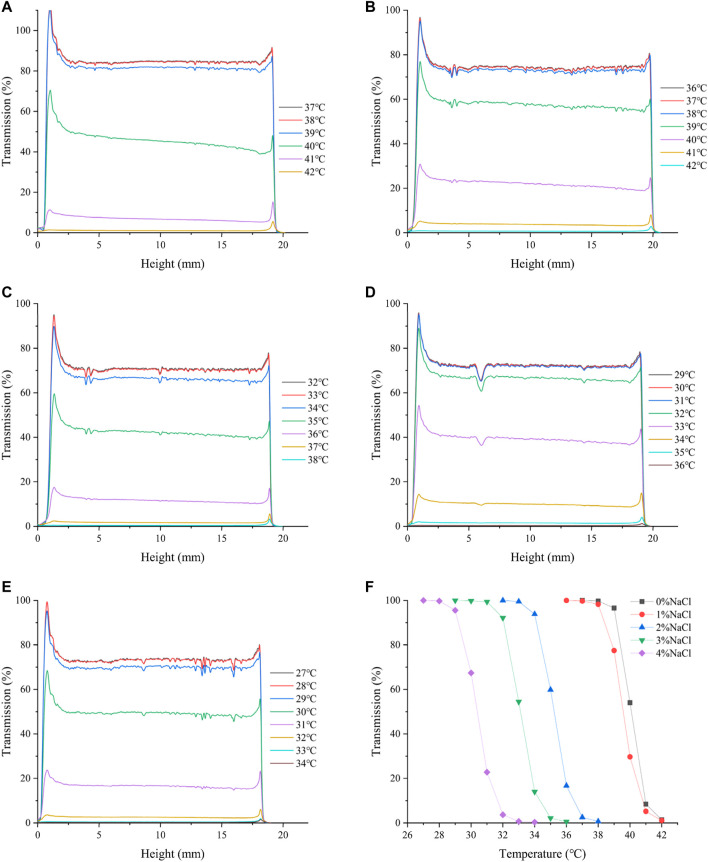
The effect of NaCl concentration on the transmission intensity profiles of HBCOS-20. **(A)** 0%, **(B)** 1%, **(C)** 2%, **(D)** 3%, **(E)** 4%. **(F)** Normalized transmission intensity of HBCOS-20.

Figure 6 shows the effect of NaCl concentration on the phase transition of HBCOS-20. It was obvious that the addition of NaCl could result in the lower LCST of HBCOS-20. Particularly, as the NaCl concentration increased to 4%, the LCST decreased to 30°C (Table 3). Similar results were reported in 2-hydroxy-3-isopropoxypropyl starch and PNIPAM solutions (Van Durme et al., 2005; Zhang et al., 2007; Ju et al., 2013). These phenomena could be explained by the stronger interaction between NaCl and water molecules than the interaction between HBCOS and water molecules. The addition of NaCl destroyed a portion of hydrogen bonds between HBCOS and water, and decreased the solubility of HBCOS, resulting in a decrease in the LCST of HBCOS solutions (Van Durme et al., 2005). The increased surface tension induced by NaCl may be another reason for the lower LCST (Tatar Guner and Demirel, 2012).

**TABLE 3:** Influence of NaCl concentration on the LCST and particle size of HBCOS-20.^†^

NaCl concentration (w/v)	LCST (°C)	Diameter (nm)	
		Before phase transition	After phase transition
0	40.20 ± 0.42^e^	3.46 ± 0.78	663.31 ± 33.51
1	38.50 ± 0.58^d^	2.81 ± 0.58	672.02 ± 47.05
2	35.50 ± 0.58^c^	2.78 ± 0.11	670.32 ± 32.70
3	32.75 ± 0.96^b^	2.21 ± 0.28	609.80 ± 19.94
4	30.00 ± 1.00^a^	3.58 ± 0.58	473.04 ± 34.65

† The different superscript letters within the same column indicate significant difference (*p* < 0.05).

### Thermoresponsive Mechanism of Hydroxybutyl Chitosan Oligosaccharide

**FIGURE 7:**
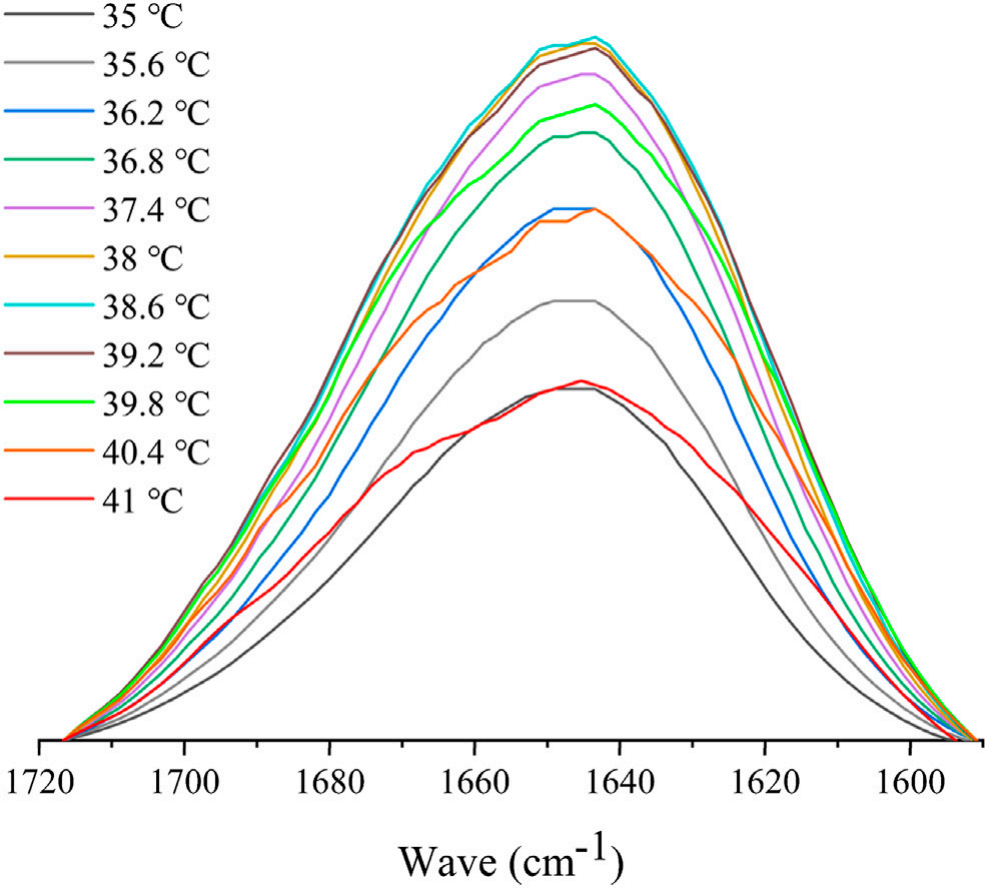
FTIR spectral variations in the amide I region of 10 mg/ml HBCOS-20 as a function of temperature.

Variable-temperature FTIR spectroscopy was used to explore the changes in the chemical groups in interaction and conformation during the polymer phase transition (Lai and Wu, 2010). To prevent the δ(OH) band of water from overlapping with the amide I band, D_2_O was used in this study as the solvent for FTIR measurements as opposed to water (Maeda et al., 2000; Sun et al., 2008). The IR spectral variations of HBCOS-20 in the C–H and C=O regions induced by temperature are shown in Figure 7 and Supplementary Figure S3. The absorption peaks at 2,965, 2,932, and 2,878 cm^−1^ were attributed to ν_as_ (CH_3_), ν_as_ (CH_2_), and ν_s_ (CH_3_), respectively. As the temperature increased, the bands of ν_as_ (CH_3_), ν_as_ (CH_2_), and ν_s_ (CH_3_) were unchanged (Supplementary Figure S3). However, the C–H stretching bands of HBCOS in D_2_O were close to the measurement of neat solid state (Figure 1). This phenomenon indicated that the alkyl groups of HBCOS were already dehydrated in the range of test temperature (Maeda et al., 2002). The dehydrated hydrophobic group could occur hydrophobic association, resulting in the aggregation or collapse of HBCOS. Similar results have been reported in PNIPAM and poly (3-ethyl-*N*-vinyl-2-pyrrolidone) (Sun et al., 2008; Lai and Wu, 2010; Lai et al., 2012), which revealed that the dehydration of hydrophobic chains of HBCOS played a role in the phase transition. The intensity of the amide I band at 1,647 cm^−1^ increased initially as the temperature increased, then decreased when the temperature was above the LCST. Meanwhile, a new peak at 1,670 cm^−1^ also appeared, and the band shape of amide I changed from a relatively symmetrical form to an asymmetrical form (Figure 7). The absorption peaks at 1,647 and 1,670 cm^−1^ were attributed to the hydrogen bonds formed between the C=O groups with D_2_O and DN, respectively. These results indicated that a portion of the C=O groups dehydrated and formed new hydrogen bonds with the DN group during the phase transition. The conversion of hydrogen bond types from C=O•••D–O–D to C=O•••N–D also contributed to the phase transition of HBCOS (Sun et al., 2008; Lai et al., 2012). Overall, the dehydration of hydrophobic chains and the transition of hydrogen bonds within hydrophilic groups (amide I region) were the driving forces for the phase transition of HBCOS.

### Cytotoxicity of Hydroxybutyl Chitosan Oligosaccharide

**FIGURE 8:**
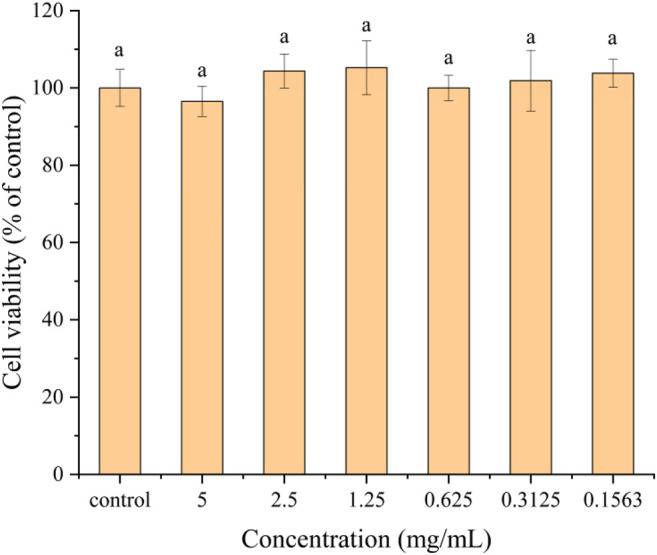
Cytotoxicity of HBCOS-20 solution with different concentrations to Caco-2 cells after 24 h incubation. Different letters above bars indicate significant differences (*p* < 0.05).

The cytotoxicity of HBCOS with different concentrations toward Caco-2 cells was determined by CCK-8 assay. As shown in Figure 8, there was no significant difference in cell viability between the control cells and cells treated with different concentrations of HBCOS. This result revealed that the HBCOS were noncytotoxic. The satisfactory cytocompatibility of HBCOS showed enormous potential for biomedical applications.

## Conclusion

In this paper, the thermosensitive HBCOS was generated by the alkylation modification of COS with 1,2-epoxy butane. The modification mainly occurred at the C_6_–OH position in COS, and the DS was controlled by the adding volume of 1,2-epoxy butane. The LCST of HBCOS was negatively correlated with the DS, HBCOS solution concentration, and NaCl concentration. The solution-to-nanogel transition of HBCOS induced by temperature was caused by the dehydration of the hydrophobic chains and conversion of hydrogen bond types. Moreover, the noncytotoxicity of HBCOSs was proven by *in vitro* cytotoxicity assay. Our research provides a regulable thermosensitive hydroxybutyl chitosan oligosaccharide, which possesses enormous potential applications in food, medicine, and biomedical fields.

## Data Availability

The original contributions presented in the study are included in the article/Supplementary Material, further inquiries can be directed to the corresponding authors.
