# Supplementary material for: Tunable Thermo-Responsive Properties of Hydroxybutyl Chitosan Oligosaccharide
Source: Front Chem. 2022 Mar 10;10:830516. doi: 10.3389/fchem.2022.830516 (PMC8960259; doi:10.3389/fchem.2022.830516)
Supplement: Supplementary file 1 [file DataSheet1.docx]

Supplementary Material


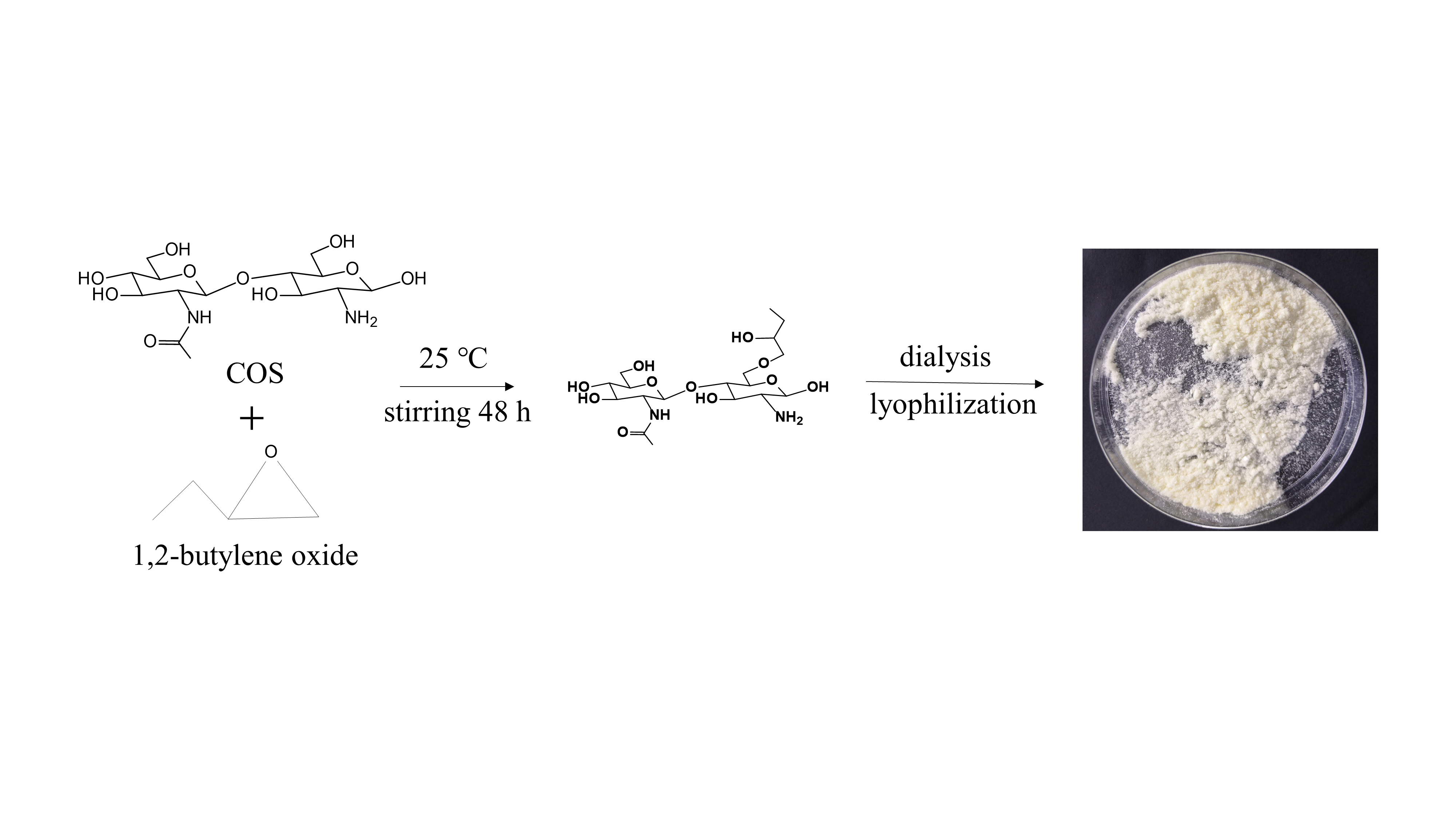


Fig. S1. Synthetic procedure of HBCOS





Fig. S2. Changed in size distribution of HBCOSs before (Dotted line) and after (solid line) phase transition.





Fig. S3. Changes of FTIR spectra in the C-H region of 10 mg/mL HBCOS-20 as a function of temperature.
